# The establishment of a bank of stored clinical bone marrow stromal cell products

**DOI:** 10.1186/1479-5876-10-23

**Published:** 2012-02-06

**Authors:** Marianna Sabatino, Jiaqiang Ren, Virginia David-Ocampo, Lee England, Michael McGann, Minh Tran, Sergei A Kuznetsov, Hanh Khuu, Arun Balakumaran, Harvey G Klein, Pamela G Robey, David F Stroncek

**Affiliations:** 1 Department of Transfusion Medicine, Clinical Center, National Institutes of Health, Building 10, Room 1C711, Bethesda, MD 20892-1184, USA; 2 Craniofacial and Skeletal Disease Branch, National Institute of Dental and Craniofacial Research, National Institutes of Health, Bethesda, MD, USA

## Abstract

**Background:**

Bone marrow stromal cells (BMSCs) are being used to treat a variety of conditions. For many applications a supply of cryopreserved products that can be used for acute therapy is needed. The establishment of a bank of BMSC products from healthy third party donors is described.

**Methods:**

The recruitment of healthy subjects willing to donate marrow for BMSC production and the Good Manufacturing Practices (GMP) used for assessing potential donors, collecting marrow, culturing BMSCs and BMSC cryopreservation are described.

**Results:**

Seventeen subjects were enrolled in our marrow collection protocol for BMSC production. Six of the 17 subjects were found to be ineligible during the donor screening process and one became ill and their donation was cancelled. Approximately 12 ml of marrow was aspirated from one posterior iliac crest of 10 donors; one donor donated twice. The BMSCs were initially cultured in T-75 flasks and then expanded for three passages in multilayer cell factories. The final BMSC product was packaged into units of 100 × 10^6 ^viable cells, cryopreserved and stored in a vapor phase liquid nitrogen tank under continuous monitoring. BMSC products meeting all lot release criteria were obtained from 8 of the 11 marrow collections. The rate of growth of the primary cultures was similar for all products except those generated from the two oldest donors. One lot did not meet the criteria for final release; its CD34 antigen expression was greater than the cut off set at 5%. The mean number of BMSC units obtained from each donor was 17 and ranged from 3 to 40.

**Conclusions:**

The production of large numbers of BMSCs from bone marrow aspirates of healthy donors is feasible, but is limited by the high number of donors that did not meet eligibility criteria and products that did not meet lot release criteria.

## Background

Bone marrow-derived stromal cells (BMSCs) are adult multipotent cells that can be isolated from bone marrow [1,2]. For their multitude of actions they represent a very attractive tool in cellular therapies; osteogenesis imperfecta [3,4], acute and chronic graft versus host disease (GVHD) [5-11] , inflammatory bowel disease [12], ischemic heart disease [13], non-healing ulcers [14], ischemic stroke [15], multiple sclerosis [16], amyotrophic lateral sclerosis [16,17], Parkinson's disease [18] , and spinal cord injury [19]. These are only a few examples of their application in phase I, II and III clinical trials.

Traditionally, BMSCs are derived from marrow aspirates or marrow tissue associated with surgical bone specimens or bone biopsies, but the percentage of marrow cells which are BMSCs is very low; between 0.01-0.001%. For in vivo use, BMSCs must be expanded to reach adequate numbers for therapeutic doses. For this reason cell processing facilities have established processes for the large scale production of BMSCs for autologous use when long-term BMSC engraftment and differentiation may be required and the use of HLA-matched BMSCs is needed [20-22]. However, when long-term survival of BMSCs is not necessary, the use of BMSCs without regard for HLA-matching has been shown to be effective. The effectiveness of third party donor BMSCs is likely due to the modulation of the host immune and inflammatory response by cytokines and growth factors released by BMSCs. For these applications banks of third party human BMSCs have been created where BMSCs are isolated, expanded ex vivo over several weeks, cryopreserved and finally thawed and administered to patients determined eligible for specific treatments [5,6,10].

In 2008 the NIH Bone Marrow Stromal Cell Transplantation Center (BMSC TC) was established. The aim of the center was to create the infrastructure to manufacture clinical grade human BMSCs and to facilitate the use of ex vivo expanded BMSCs for the treatment of patients with a variety of human diseases and disorders within the Clinical Center. In this paper we report the manufacturing process we have optimized and validated to produce "clinical grade" BMSCs in support of the BMSC TC activities. The processes used to screen donors, collect marrow, and to produce and cryopreserve BMSCs are described as well as the results of the first 11 full scale BMSC production runs in our GMP facility.

## Methods

### Donor eligibility and donor screening process

All donors were required to meet Food and Drug Administration (FDA) and AABB (formally the American Association of Blood Banks) criteria for cellular therapy donors. A protocol for the donation of marrow for BMSC production was created, posted on the NIH website and registered in clinicaltrials.gov with the identification number NCT01071577. Potential volunteers made the initial contact with the Department of Transfusion Medicine (DTM, NIH) by phone or email and the protocol procedures and risks were explained. Demographic information, an abbreviated questionnaire for determination of risk factors for transmissible infections including recent travel outside of the USA or Canada and an abbreviated medical history were obtained. If no factors were identified that would make him/her ineligible, they were then invited to make a follow-up appointment in the Donor Center for a complete donor eligibility evaluation. At this point a NIH healthy volunteer consent was obtained and a unique NIH medical record number (MRN) assigned and legal identification verified to insure correct patient identity and age since the donor's age must be greater than or equal to 18 years. In a private setting a thorough explanation of the protocol including risks was completed, time was allowed for donor questions, and then informed consent obtained. Three forms, AABB donor education materials, medical deferral list, and foreign travel information sheet (a list of medications that cause deferral and a list of the countries in Europe and the United Kingdom where travel or residency can result in a permanent deferral from donating due to variant Creutzfeldt-Jakob disease (vCJD) risk) were reviewed. An FDA approved standardized screening questionnaire for cellular therapy products was administered verbally by trained DTM staff and answers were recorded on paper. This questionnaire documents an abbreviated medical history and screens for risk factors that identify a donor's potential for exposure to HIV, hepatitis, and other viral infections as well as exposure to malaria, vCJD and other diseases. This paper became part of the official record that is kept in a secure folder in a locked room.

#### Donor testing

A brief physical exam was performed; blood samples were collected and tested for markers of transfusion transmitted diseases (TTD), complete blood counts (CBC), coagulation assays, HLA type, ABO blood group and pregnancy where appropriate. Donors were tested for anti-HIV-1/2, hepatitis B virus surface antigen (HBs Ag), anti-hepatitis B virus core antigen (anti-HBC), anti-hepatitis C virus (anti-HCV), RPR-*Treponema pallidum *(syphilis), anti-HTLV-1, anti-HTLV-2, cytomegalovirus IgG, IgM antibody (anti-CMV), anti-*T. Cruzi *(Chagas), HIV-1/HCV/HBV nucleic acid testing (NAT) and West Nile Virus NAT. Donors were also tested for CBC with differential white cell count, prothrombin time (PT), partial thromboplastin time (PTT) and HLA- A, B, C and DR/DQ. With the exception of the RPR, the TTD tests were sent to an outside FDA approved laboratory for donor screening assays. The donor must have a platelet count > 150 × 10^9^/L, absolute neutrophil count > 1.0 × 10^9^/L and hemoglobin > 11.5 g/dL for African or African American women, > 12.0 g/dL for all other women and 12.5 g/dL for all men and the PT and PTT must be within normal limits. The results of the TTD, CBC, PT, PTT and pregnancy assays were used to determine donor eligibility while the results of ABO blood group and HLA type were used for identity testing of the product.

If the potential donor met the eligibility criteria, the marrow aspiration was scheduled. This was done within 30 days of the pre-screening visit according to AABB and FDA standards.

### Bone marrow collection

On the day of marrow aspiration, the donors first reported to the Blood Donor Center and were registered into our center's Blood Bank Computer System (BBCS, Blood Bank Computer Systems Inc, Auburn, Washington), a password protected data management system that assigns each donor a unique donor identification number and tracts the results of donor screening via a Self Reporting Questionnaire (SRQ) as well as physical examination, ABO grouping and transfusion transmitted disease tests. After the donor was registered an abbreviated physical exam was repeated and another blood sample was collected for TTD testing and ABO grouping as previously reported.

The donor was then taken to a hospital procedure room. First, the risks of bone marrow aspiration were reviewed and a surgical consent was signed by each donor. As required by the Joint Commission on Accreditation of Healthcare Organizations (JCAHO), a "time out" was taken to verify donor identity and donor understanding of the procedure to be performed. Bone marrow aspirates were collected according to the standard procedure in use at the NIH Clinical Center; the protocol allows for bilateral collection, but to date unilateral aspirates only have been collected.

A 1 mL aliquot of bone marrow at each collection was evaluated by a hematopathology medical technologist in the Department of Laboratory Medicine for the presence of bone spicules as a measurement of aspirate quality; a marrow smear and peripheral blood smear is reviewed as well for normal trilineage hematopoiesis. A limited volume of marrow was aspirated (max 12 mL) to avoid excessive dilution with peripheral blood, and collected in multiple 3 mL Bone Marrow Prep Syringes (Pharmacy Department, CC, NIH) containing DMEM, heparin and gentamicin. Syringes were properly labeled and transported at room temperature to the CPS laboratory.

### Marrow processing and BMSC production

#### Receipt of marrow by the cell processing laboratory

The marrow aspirate was received by trained cell processing laboratory staff. At the time of receipt each syringe was inspected and its appearance, identifiers and time of receipt was documented. The samples were processed immediately, but the laboratory's procedures allow samples to be stored at 2-8°C for a maximum of 4 h prior to processing.

#### Overview of processing

The BMSCs were initially cultured in T-75 flasks. They were then expanded for three passages in multilayer cell factories. At the completion of the processing the BMSCs from the final harvest were packaged into units of 100 million viable cells, cryopreserved and stored in the vapor phase of a liquid nitrogen storage tank.

#### Marrow aspirate receipt and evaluation

After receipt, the aspirates were pooled into a 50 mL conical tube (BD Falcon, BD Biosciences, Bedford, MA) and total volume estimated and diluted up to 3.5 times by adding Bone Marrow Stromal Cell Culture Media (BMSC CM) which consisted of: alpha MEM (Lonza, Walkersville, MD) supplemented with 20% lot-selected US origin Defined Fetal Bovine Serum (FBS) (Hyclone Laboratories, Inc., Logan, UT) and gentamicin sulfate (10 mcg/mL) (Gentamicin, injection, UPS, AAPP Pharmaceuticals, LCC, Schaumburg, IL). A single cell suspension was prepared; cells were passed through an 18 ga spinal needle (18 ga, 3.5 in., Monoject, Covidien, Mansfield, MA) twice and then through a 20 ga spinal needle (BD Spinal Needle, BD Medical, Franklin Lakes, NJ) twice and resuspended in 20 to 30 mL with BMSC CM.

A 1 mL aliquot of solution was removed for cell counts (Celldyne 3700, Abbott Laboratories Inc., Abbot Park, IL), and analysis by flow cytometry for CD3 and CD34 content and viability using 7AAD (BD FACSCanto, BD Biosciences, San Jose, CA) (Table 1). An in-process control was set at this point and the aspirate was discarded if the total nucleated cell (TNC) count was < 20 × 10^6^. Colony Formation Efficiency (CFE) was performed as previously described [23] as a quality measure of the starting material. The CFE and CD34+ cell counts were obtained for retrospective analysis, but were not used to qualify the starting material.

**Table 1:** BMSC lot release criteria

Test	Method	Criteria
CD73	Flow cytometry	≥80% reactive cells
CD90	Flow cytometry	≥80% reactive cells
CD105	Flow cytometry	≥80% reactive cells
CD146	Flow cytometry	≥60% reactive cells
CD34	Flow cytometry	≤5% reactive cells
CD45	Flow cytometry	≤5% reactive cells
CD14	Flow cytometry	≤5% reactive cells
CD11b	Flow cytometry	≤5% reactive cells
Viability	Trypan blue	≥70%
Sterility	Bactec Plus, aerobic and anaerobic	No growth after 14 days
Mycoplasma	PCR	Negative
Endotoxin	Limulus Amebocyte Lysate (LAL)	< 5.0 EU/mL

#### Initial growth of BMSCs

The BMSCs were initially grown in T-75 flasks (75 cm^2 ^flask, canted neck, nonpyrogenic, sterile polystyrene, Corning Incorporated, Corning, NY). The flasks were seeded at a cell density of 2 to 3 × 10^5 ^cells per cm^2 ^of surface area and a final volume of 20 mL BMSC CM. The flasks were incubated at 37°C in 5% CO_2 _and 90 ± 5% humidity. Thirty percent of the flasks were used to assess colony formation and confluence periodically during the culture by evaluating 40% of the surface of the bottom of the flasks.

On day 1 of culture, 24 h after plating the cells, supernatants containing non-adherent cells were removed through complete media exchange and the flasks re-incubated at 37°C in 5% CO_2 _and 90 ± 5% humidity. Thereafter, complete media exchange was performed every 3 days. On day 7 the flasks assigned for colony formation assessment were inspected using an inverted microscope and under 25× magnification. If no colonies were detected, the cultures were discontinued; otherwise, the cultures were maintained. Beginning on day 10 the number of colonies with more than 50 cells and > 70% confluence and with more than 50 cells and < 70% confluence was recorded. If more than half the colonies were > 70% confluent, the cells were harvested. If less than half the colonies were > 70% confluent, then BMSC CM media was replaced and the process repeated the next day. Colony counting and confluence assessment continued daily until the harvest criteria were met or the culture reached Day 13; the cultures were terminated if the criteria were not met.

Primary culture was harvested by trypsinization. We used commercially available recombinant trypsin (TryPLE Express Invitrogen, CA) to limit the usage of animal derived reagents. An aliquot of the final harvested cells was assessed for manual cell counts and viability by the trypan blue exclusion method and sterility (BD BACTEC plus + aerobic/F and BD BACTEC plus + anaerobic/F, Becton Dickenson and Company, Sparks, MD). The BMSCs were also evaluated by flow cytometry for the expression of BMSC surface markers according to the ISCT panel. The culture was discontinued if viability was < 70% or sterility testing was positive. The isolated BMSCs were designated passage 1 cells.

#### In vitro expansion

Passage 1 BMSCs were seeded in 2-layer cell factories (Cell Factory, easy fill 2-trays, Nunc A/S, Roskilde, Denmark) at a cell density of 2,500-4,000 cells/cm^2^. The plating density was based on published data. A maximum of 4 culture vessels were seeded for each processing event due to the maximum capacity of the GMP processing suite dedicated to BMSCs production. To facilitate the handling of cultures a peristaltic pump using an adaptor specifically designed was used for media and cell loading (Fluid transfer tube set, Baxa Corporation, Englewood, CO). The media levels were equalized on the two layers and the cell factories were incubated at 37°C with 5% CO_2 _and 90 ± 5% humidity.

On day 3 complete media exchange was performed. Cells were harvested on day 5 or 6 once the culture met the criteria defined as 70% confluence. Culture was discarded if confluence was < 70% on day 6. Harvest was performed as for flasks using TryPLE express. If the volume of the cells recovered was less than or equal to 1200 mL, it was reduced using a floor model centrifuge (Sorvall RC3, DuPont, Newtown, CT) set at 406 g for 10 mins. If the volume was > 1200 mL a cell washer (Cobe 2991 cell processor, Caridian BCT, Lakewood, CO) was used. The passage 2 BMSCs were resuspended in 50 mL of BMSC CM and 0.5 mL was removed for manual cell counts and viability assessment using trypan blue staining. The culture at this point was terminated if the viability was < 70%.

### BMSC passage 2 culture

Passage 2 BMSCs were seeded in 10-layer cell factories (Cell Factory, easy fill 10-trays, Nunc A/S) at a cell density of 2,500-4,000 cells/cm^2^. After measuring the number of BMSCs harvested, the number of 10-layer cell factories, each of which has a surface area of 6,300 cm^2^, that could be seeded and the number of cells to place in each cell factory was calculated. Our protocol permits a maximum of four 10-layer cell factories to be seeded with passage 2 cells. Approximately 20 × 10^6 ^per 10-layer cell factory in a final volume of 2.5 L BMSC CM per container was loaded using the automatic system described above. The media levels were equalized and cell factories incubated at 37°C with 5% CO_2 _and 90 ± 5% humidity. On culture day 3, a complete media exchange was performed, and a volume of 2.5 L fresh BMSC CM was added. The cell harvest was planned for day 5 or 6 according to the degree of cell confluence. The culture was discontinued if cell confluence was < 70% on day 6.

Harvested passage 3 cells were assessed for cell count and viability. The harvested BMSCs were concentrated using a cell washer (Cobe 2991 Cell Processor) and resuspended in 100 mL of BMSC CM.

### BMSC passage 3 culture

For passage 3 culture, 10-layer cell factories were seeded, incubated and monitored as described for passage 2, however, up to eight 10-layer cell factories were seeded. The 10-layer cell factories were also harvested on Day 5 or 6 according to confluence and the same criteria for selecting the harvest day was used. For this step HBSS with 5% heat inactivated AB plasma (HIAB) was used to inactivate the trypsin instead of BMSC CM to reduce residual level of bovine contaminants.

The final product was concentrated and washed thoroughly using a cell washer (Cobe 2991 cell processor). After loading the cells into the cell processor, they were washed for 5 cycles with Plasmalyte A (Baxter Healthcare Corporation, Westlake Village, CA) containing 0.5% HSA (Fexbumin 25% (Human) USP, 25% solution, Baxter Healthcare Corporation). The washed cells were then resuspended in Plasmalyte A with 4% HSA.

The harvested passage 4 BMSCs were counted manually and assessed for viability by the trypan blue exclusion method, and for safety with endotoxin, sterility, and mycoplasma by PCR analysis. Cell surface marker expression was assessed by flow cytometry. The cells were discarded if they did not meet lot release criteria (Table 1). The number of aliquots of 100 × 10^6 ^viable BMSCs that could be cryopreserved was calculated and the cells were suspended in a volume of 10 mL of plasmalyte A with 4% HSA for each 100 × 10^6 ^unit of viable cells.

### Cryopreservation and storage

A total of 10 mL of BMSCs at 10 × 10^6 ^cells per mL were mixed with 10 mL of freeze mix consisting of 10% DMSO, 12% Pentastarch and 8% Human Serum Albumin (HSA) in plasmalyte A and transferred into customized 20 mL FEP cryobags (AFC Kryosure VP-20f, Gaithersburg, MD, USA). The cells were cryopreserved using a controlled rate freezer (Kryosave, Integra, Planer plc, Sunbury-on-Thames, UK) and stored in the vapor phase of a liquid nitrogen tank. Each cryobag with 100 × 10^6 ^BMSCs was considered a "unit" of BMSCs. A specific label was applied according to ISBT standards.

### Quality assurance systems

A specific software program for capturing cellular therapy data (StemLab, STEMSOFT Software Inc., Vancouver, British Columbia, Canada) was used in the process described above. All components used in the manufacturing process were taken into account and recorded for further tracking: equipment, starting cellular material, ancillary reagents, materials, product inventory, personnel and methods.

### Regulatory considerations

All marrow was collected and BMSCs produced under a NHLBI IRB approved protocol and a DTM, CC, NIH Drug Master File. BMSC products are available for administration to Clinical Center patients who are enrolled in NIH intramural program IRB approved treatment protocols. All patients must also be treated under an Investigational New Drug (IND) application.

### Statistical analysis

The values shown are the mean ± 1 standard deviation (SD). The correlation coefficients and p-values were calculated by a regression model. The significance between 2 groups was evaluated by student *t*-test. *P *< 0.05 was considered significant. All the plots and statistical analysis were completed by using Microsoft Excel 2007.

## Results

Marrow was collected from 10 healthy donors. In order to identify these 10 donors a total of 17 donors were enrolled into the marrow aspiration protocol and were evaluated at our center. Of these 17 potential donors, 6 were found to be ineligible during the screening process and one developed an illness immediately prior to the scheduled donation (Table 2). A 49-year-old male was ineligible because of a history of a prolonged stay in a vCJD risk area, a 31-year-old female because of a low hemoglobin level and a 52-year-old male because of a reactive TTD test. A 26-year-old female was ineligible due to a history of an event that placed her at risk of a TTD, a 30-year-old woman had an elevated PTT result and a 25-year-old female had evidence of an acute CMV infection. A 24-year-old female developed an acute illness the day of the scheduled donation and was not allowed to donate. The remaining 10 donors met donor eligibility criteria and marrow was collected once from 9 donors and twice from one donor, donor 1. These 10 donors ranged in age from 21 to 67 years and 7 were male (Table 2).

**Table 2:** Characteristics of healthy subjects agreeing to donate marrow for BMSC production and the aspirated marrow

Donor				Aspirated Marrow					
	**Age (years)**			Volume* (mL)	**TNC (10^6^)**	**CD34**		**CD3**	
**Number**		**Gender**	**Race**			**(%)**	**#(10^6^)**	**%**	**#**

1	35.6	M	AA	21	165	2.26	3.73	10.7	17.7
2	22.9	M	W	26	266	1.56	4.15	11.7	31.1
3	22.8	F	W	27.5	374	1.20	4.48	13.3	49.7
4	67.9	F	W	27	127	0.71	0.90	5.19	6.59
5	21.7	M	W	25	584	0.66	3.85	7.62	44.5
6†	49.5	M	W	vCJD risk	NA	NA	NA	NA	NA
7	23.7	F	W	24.8	788	1.71	13.5	6.89	54.3
8†	52.1	M	W	+ TTD test	NA	NA	NA	NA	NA
9†	31.5	F	AA	Low Hgb	NA	NA	NA	NA	NA
10	27.2	M	AA	27	681	1.44	1.44	6.81	46.4
11	56.8	M	W	26	228	1.4	3.19	9.38	21.4
12†	24.9	F	W	Sick	NA	NA	NA	NA	NA
13†	26.6	F	AA	TTD risk	NA	NA	NA	NA	NA
1ŧ	36.5	M	AA	25.6	153	1.99	3.04	10.4	15.9
14	21.3	M	AA	27	271	1.45	3.93	13.5	36.6
15	59.4	M	W	25	160	0.45	0.72	8.67	13.9
16	25.2	F	W	+CMV IgM	NA	NA	NA	NA	NA
17†	30.8	F	A	Elev. PTT	NA	NA	NA	NA	NA

*Volume volume of media plus volume of aspirated marrow

† Ineligible to donate

Ŧ marrow was collected from donor 1 twice

*NA *not applicable

*M *male; *F *female

*AA *African American; *W *White

*TTD *transfusion transmitted disease

The total volume of aspirated marrow plus media in the 8 syringes obtained from each donor ranged from 21 to 27 mL. The total number of nucleated cells collected was 345 ± 233 × 10^6 ^(mean 1 ± SD) and ranged from 127 × 10^6 ^to 788 × 10^6^; the average proportion of cells expressing CD34 was 1.35 ± 0.56% and ranged 0.45% to 2.26%. Bone marrow spicules were present in all aspirates except those from donor 4. All marrow aspirate and peripheral blood smears were within normal limits.

For all 11 donations, BMSC colonies were detected in T-75 flasks in the primary culture at day 7. For marrow aspirates from donors 4 and 15, an insufficient number of colonies were detected on day 7 to continue on the culture. For the second marrow aspirate from donor 1, during the culture of BMSCs in 2-layer factories, post-donation information became known that made the donor ineligible and the culture was discarded. The other 8 donations were successfully cultured through passage 4 and the cells were harvested (Table 3). The final yield of BMSCs ranged from 990 × 10^6 ^to 4760 × 10^6 ^resulting in 9 to 40 BMSC units. BMSC products from 7 of the 8 donations met all lot release criteria including sterility testing, viability testing, and flow marker analysis (Table 4). The expression of CD34 antigen by BMSCs from donor 15, 5.6%, exceeded the lot release criteria of ≤5%.

**Table 3:** Number of cells harvested, number of population doublings and doubling time for each BMSC passage

		T-75 flasks		2-layer cells factories			First culture in 10-layer cell factories			Second culture in 10-layer cell factories				
**Donation Number**	**Donor**	**CFE**	**Yield (×10^6^)**	**Yield (×10^6^)**	**Population Doublings**	**Doubling Time (hr)**	**Yield (×10^9^)**	**Population Doublings**	**Doubling Time (hr)**	**Yield (×10^9^)**	**Population Doublings**	**Doubling Time (hr)**	**Cumulative Population Doublings**	**Units in Storage**

W092110086001	1	6	6.5	94.4	3.9	30.9	0.81	3.2	37.3	1.73	3.4	34.9	10.5	10
W092110086002	2	18	30.3	372	4.5	26.4	0.99	3.6	33	2.43	3.9	30.6	12	19
W092110086003	3	9	21	314.3	4.3	27.9	1.28	4	30	1.61	3.3	36	11.6	14
W092110086004	4	0	0.2	NA	NA	NA	NA	NA	NA	NA	NA	NA	NA	NA
W092110086005	5	11.5	52.1	456.4	4.8	24.8	1.23	3.9	30.4	4.76	4.9	24.5	13.6	40
W092110086006	7	15	28	372.5	4.5	26.4	0.9	3.5	34.3	2.17	3.8	31.9	11.8	20
W092110086007	10	1.5	25.4	255.4	4	30	1.1	3.8	31.8	1.66	3.4	35.6	11.2	15
W092111086001	11	7.5	3.9	27.9	3.9	30.9	0.15	2.9	49.8	0.99	3	47.2	8.8	9
W092111086002	1	7	3.1	NA	NA	NA	NA	NA	NA	NA	NA	NA	NA	NA
W092111086003	14	5.5	12.9	233.2	4.3	28	0.97	3.6	33.3	2.52	3.98	30.2	11.9	23
W092111086004	15	4.5	2.2	NA	NA	NA	NA	NA	NA	NA	NA	NA	NA	NA

*NA *not applicable; *CFE *colony formation efficiency

**Table 4:** Characteristics of the 8 lots of BMSCs Produced

	Biomarker Expression (Percent Reactive Cells)										
				
Donor	CD73	CD90	CD105	CD146	CD34	CD45	CD14	CD11b	Viability	Sterility	Endotoxin (EU/mL)
1	99.0	99.0	100.0	96.9	1.6	1.7	1.0	0.9	89.0	No Growth	< 5.0
2	100.0	100.0	100.0	99.5	0.5	0.6	0.1	0.6	89.0	No Growth	< 5.0
3	99.8	99.7	99.8	95.1	2.3	1.2	0.2	1.1	92.0	No Growth	< 5.0
5	99.9	99.9	99.9	99.9	2.2	0.9	0.7	0.9	95.0	No Growth	< 5.0
7	99.8	99.8	99.8	99.6	2.2	0.8	0.6	0.8	95.0	No Growth	< 5.0
10	99.7	99.7	99.8	99.7	0.9	0.9	0.6	0.9	94.0	No Growth	< 5.0
11	99.4	99.4	99.7	98.9	3.4	1.2	1.4	1.6	95.0	No Growth	< 5.0
14	99.6	99.6	99.7	98.9	5.6	0.5	0.1	0.5	94.0	No Growth	< 5.0

**Figure 1:**
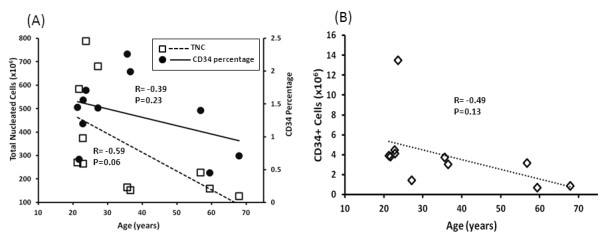
**Effects of donor age on the quantity of CD34+ cells and total nucleated cells (TNC) in the aspirated marrow**. The relationship between the age of the donor and the quantity of TNCs (Panel **A**, open squares), percentage of leukocytes expressing CD34 (Panel **A**, filled circles) and the total number of CD34+ cells in the marrow aspirate (Panel **B**) are shown. R stands for correlation coefficient and P stands for P value both of which were calculated using a regression model.

In general, TNC and CD34 percentage of marrow cells from younger donors were higher than those from older donors (Figure 1, Panel A), however the correlation was not significant (*p *= 0.06 between age and TNC; *p *= 0.23 between CD34 percentage and age). Similarly, absolute number of CD34 positive cells tended to be inversely correlated with age (*p *= 0.13, Figure 1, Panel B). Interestingly, among the 11 collections, the TNC and number of CD34 positive cells collected from the two oldest donors, 59 and 67 years old, were the lowest, and their cells failed to meet colony formation criteria at the end of the primary culture.

**Figure 2:**
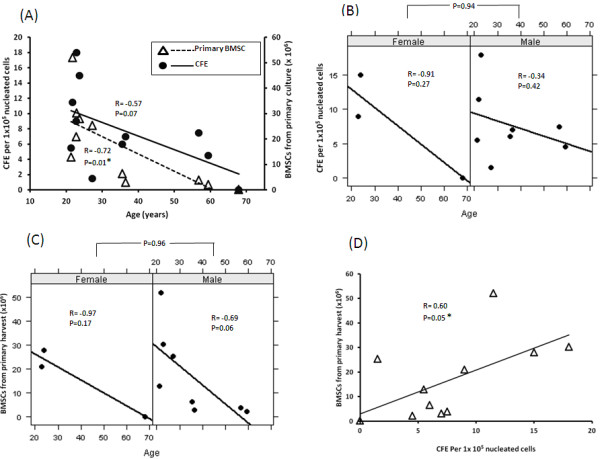
**Effect of donor age on the marrow aspirate CFE and the quantity of BMSCs harvested from the primary culture**. The relationship between the age of each donor and the CFE of the aspirated marrow (solid circles) and the quantity of BMSCs harvested from the primary culture (open triangles) is shown in panel **A**. The effects of donor age and gender on the marrow aspirate CFE is shown in panel **B **and on the quantity of BMSCs from the primary harvest is shown in panel **C**. The relationship between marrow aspirate CFE and the quantity of BMSCs from the primary harvest is shown in panel **D**. R stands for correlation coefficient and P for P value. Both were calculated using a regression model. Differences between males and females were compared using t-tests. * indicates significant differences (*p *< 0.05)

We assessed the CFE of the marrow aspirated from each donor. There was a highly significant inverse correlation between CFE and the age of donors (*p *= 0.01, R = -0.72, Figure 2, Panel A). In addition, the quantity of BMSCs harvested from primary culture tended to decrease when age increased, but the correlation was not significant (*p *= 0.07, Figure 2, Panel A). The inverse correlation between CFE and age was observed in both female and male donors, and no significance difference in CFE was observed between males and females (*p *= 0.94, Figure 2, Panel B). Similarly, the quantity of BMSCs in the primary harvest did not differ significantly between males and females (*p *= 0.96, Figure 2, Panel C). Of note, the number of BMSCs from the primary harvest increased as the CFE increased (*p *= 0.05, Figure 2, Panel D).

**Figure 3:**
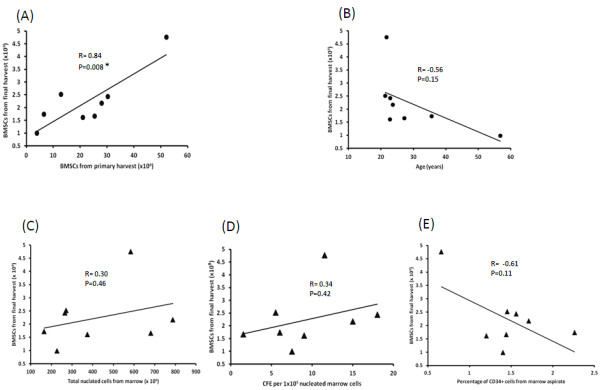
**Factors affecting the quantity of BMSCs from the final harvest**. The relationship between the quantity of BMSCs from the final harvest and the quantity of BMSCs in the primary harvest are shown in Panel **A **and the relationship between BMSCs in final harvest and donor age are shown in Panel **B**. The effects of the quantity of TNC in the marrow aspirate (Panel **C**), marrow aspirate CFE (Panel **D**) and percentage of leukocytes expressing CD34 in the marrow aspirate (Panel **E**) on the quantity of BMSCs in the final harvest are also shown. R stands for correlation coefficient and P for P value. Both were calculated using a regression model. *indicates significant differences (*p *< 0.05)

Among all the potential predictors for BMSC final harvest, the quantity of BMSCs in the primary harvest was the best one. It had a highly significant correlation with quantity of BMSCs in the final harvest (*p *= 0.008, R = 0.84, Figure 3, Panel A). The quantity of BMSCs in the final harvest decreased as donor age increased (*p *= 0.15, R = -0.56, Figure 3, Panel B), but was not affected by the marrow aspirate TNC count (*p *= 0.46, Figure 3, Panel C) or CFE (*p *= 0.42, Figure 3, Panel D). However, there was an inverse correlation between quantity of BMSCs in the final harvest and the percentage of CD34 positive cells in the marrow aspirate, although the correlation was not significant (*p *= 0.11, R = -0.61, Figure 3, Panel E).

## Discussion

We established a mechanism to manufacture and store BMSC products from healthy subjects. The BMSC bank was established to support the activity of the NIH Bone Marrow Stromal Cell Transplantation Center which involves the treatment of patients at the NIH Clinical Center who are enrolled in IRB approved treatment protocols. Our banked cells were produced from bone marrow aspirates from healthy subjects, which were plated without removal of red blood cells and expanded using progressively larger plastic surfaces up to passage 4. Although this restriction on passage number limited the quantity of BMSCs produced, it was chosen based upon data collected in preclinical studies which showed that limiting BMSC expansion to less than 40-50 population doublings minimized growth related senescence. The banked products we manufactured differ from those manufactured at some other centers in that we only produced cells from third party donors rather than both relatives of patients needing BMSC therapy and third party donors [6,10]. Our bank also does not include the storage of intermediate products for further manufacture [9,13,24]. These intermediary products are thawed and further expanded increasing their time in culture which increases the possibility of producing senescent cells, and a potentially less active final product compared to BMSCs products made up of younger cells [9,14,25].

Our established process allowed for the production of 9 to 40 doses of BMSCs from each donor. The currently approved treatment protocols at our institution involve the treatment of patients two to three times with a dose of 1 to 2 × 10^6 ^cells per kg weight. As a result approximately 2 to 9 BMSCs products are needed to treat each patient. Whenever possible each BMSC recipient is given BMSCs from a single donor or lot, so each lot could treat 1 to 20 patients. We have, however, elected to limit the use of each lot to the treatment of 1 to 4 recipients. While all donors meet cell therapy donor criteria and are tested in the same manner as whole blood, platelet apheresis donors and allogeneic cell therapy donors, there is a remote, but real possibility that BMSCs could transmit a pathogen to the recipient. Limiting the number of people treated with each lot limits the number of recipients exposed to a potential pathogen.

We found that the CFE of the aspirated marrow and the quantity of BMSCs in the primary and final harvests were less in older donors. The results are similar to previous studies which found that CFE of marrow preparations was greater in younger subjects [23]. It may be worthwhile to limit the collection of marrow for the production for BMSCs to younger donors, but sufficient data is not yet available to establish an upper age limit. We also found that the quantity of BMSCs in the final harvest was most closely related to the quantity obtained from the primary harvest.

BMSCs produced by our center have several potential advantages over the use of BMSCs from centralized or commercial facilities that produce multiple large lots of BMSCs from a single donor. Our BMSC products are earlier passage cells. The properties of BMSCs change with serial passage. Late passage BMSCs are less potent than early passage BMSCs in several assays. Their proliferation rate is less and their osteogenic and adipogenic differentiation potential is reduced [26,27]. When BMSCs are co-cultured with hematopoietic stem and progenitor cells (HPC), early passage BMSCs maintained HPC CD34 antigen expression over more cell divisions than late passage BMSCs [28]. Many studies do not provide detailed information on the production of the BMSCs used in their trials, but BMSC products from one study that successfully treated acute GVHD were passage 1 through 4 cells [10]. Another recent BMSC clinical trial suggested that the survival of therapy resistant acute GVHD patients treated with early passage BMSCs was better than those treated with late passage cells [29]. It is not certain at which passage BMSCs change from an early to late passage phenotype, but it is quite possible that BMSCs produced in large lots and that have undergone 5 or more passages may have begun to acquire a late passage phenotype and may be less effective [25].

There is also considerable evidence of variability in BMSCs due to donor factors. These factors include donor age, inherent inter-donor biological variability and day-to-day intra-donor variability. The use of a large number of BMSC products produced from only 1 or 2 donors could skew the clinical results of BMSC therapy due to donor selection. It could be that all of the BMSC products used for one clinical trial are from a single donor and all BMSC products for another similar clinical trial are from a second donor, but the outcomes of the trials differ because of differences due to donor factors. On the other hand the overall outcomes of clinical trials that make use of multiple lots of BMSC products produced from several different donors are less likely to be effected by variability of individual donor BMSCs.

While BMSCs have been shown to be effective in a number of early phase clinical trials, the critical properties of BMSCs that are responsible for their effectiveness are not known. BMSCs secrete many cytokines and growth factors and many of these may be responsible for the clinical effectiveness of BMSC products. However, it is possible that the mechanisms of action are complex and multiple factors may be responsible for their clinical effectiveness. Furthermore, the factors contributing to clinical effectiveness may vary with disease type. As a result at this time there are no high quality biomarkers for assessing BMSC potency. Due to the lack of good BMSC potency biomarkers we choose to produce BMSCs using techniques that are very similar to those used by other groups whose BMSC products have been shown to be clinically effective; growth in FBS and the use of low passage number cells. Some other production facilities have elected to use media supplemented with growth factors and cytokines rather than FBS [30,31]. However, it is not clear if these changes in BMSC production techniques will influence their clinical effectiveness.

We found that our cell processing laboratory could scale up, produce and validate BMSC products, but this process is relatively expensive. The use of healthy donors for BMSC production required the development of a number of new procedures and data collection, storage and monitoring systems. These procedures and systems required considerable time and resources to develop. However, producing BMSCs in our institution provides several benefits.

Our donor screening and evaluation, marrow collection and BMSC production process is well defined and highly controlled. By tightly controlling this process we expect to maximize consistency among the lots of BMSC products. This will help minimize variability in clinical outcomes due to lot-to-lot variability.

The use of BMSC products made within our facility permits product analysis and clinical correlation with product characteristics that are critical to the development of new biological therapies. We are storing aliquots from each BMSC lot that will be used clinically to evaluate each lot and compare BMSC properties to clinical outcomes. These studies will likely improve the understanding of the mechanism of action of BMSCs. Commercial manufacturers of BMSC products often do not permit the analysis of their BMSC products by the treating hospital or clinicians. Also, the exact nature of the commercial formulation is not known and there are many concerns on the manufacture of commercial product that may contribute to the less than optimal results. At least one of the commercially available products used bone marrow aspirates from a single unrelated donor to generate millions and millions of cells [13]. In such an instance, it is probable that growth factors were added to BMSC cultures by the company. The addition of growth factors such as fibroblastic growth factor, have been known to result in the loss of a subset of stem cells in bone marrow stromal cells [32]. The enormous number of population "doublings" in the strategy used commercially will result in telomere shortening; cells with longer telomeres were found to be critical for the success of adoptive transfer of tumor infiltrating T lymphocytes in patients with metastatic melanoma [33].

Producing BMSCs at our institution also allows us to modify our production process if an alternative method that results in a more effective product is identified at our institution or at another institution. If we had elected to use cells produced by a commercial laboratory, we would have no control over the production of the cells.

## Conclusions

To our knowledge this is the most comprehensive description of a GMP program at an academic health center to assess third party BMSC donors, aspirate marrow, produce and store BMSC products for clinical use. The GMP production and storage of clinical BMSCs from healthy third party donors is feasible, but is limited by the high number of donors that did not meet eligibility criteria and products that did not meet lot release criteria.

As BMSC therapy matures mechanisms of action will be defined and potency markers identified and the best production method described. At that time for many clinical applications it will likely be best to produce BMSCs at large commercial facilities. However, in this early stage of the development of this field, the production of BMSCs in academic facilities is beneficial and in many ways preferred.

## Competing interests

The authors declare that they have no competing interests.

## Authors' contributions

MS led the development and scale up of the GMP BMSC culture methods, analyzed the data and wrote the manuscript. JR developed and performed the large scale BMSC culture methods, analyzed data, prepared figures and helped write the manuscript. VD-O helped develop GMP culture and processing methods and helped establish clinical BMSC manufacturing. LE developed methods for screening and assessing donors and for collecting marrow. In addition, LE collected the marrow samples, prepared tables and helped write the manuscript. MT led the production of the GMP manufacturing of the BMSC lots. SAK helped with the scale up of the methods for culturing BMSCs and helped write the manuscript. HK helped develop the methods for the GMP manufacturing of BMSCs. AB assisted with the scale up of the BMSC culture methods and helped write the manuscript. MM developed the computer applications for documenting donor eligibility and product manufacturing, storage, release and issue and helped write the manuscript. HGK, PGR and DFS helped with the development of all aspects of the program. DFS also assisted with the marrow collections, analyzed the data and wrote the manuscript. All authors read and approved the final manuscript
